# The heavy metals lead and cadmium are cytotoxic to human bone osteoblasts via induction of redox stress

**DOI:** 10.1371/journal.pone.0225341

**Published:** 2019-11-22

**Authors:** Ayat Al-Ghafari, Ekramy Elmorsy, Emad Fikry, Majed Alrowaili, Wayne G. Carter

**Affiliations:** 1 Biochemistry Department, Faculty of Science, King AbdulAziz University, Jeddah, Saudi Arabia; 2 Department of Forensic Medicine and Clinical Toxicology, Faculty of Medicine, Mansoura University, Mansoura City, Egypt; 3 Department of Pathology, Faculty of Medicine, Northern Border University, Arar; Saudi Arabia; 4 School of Medicine, University of Nottingham, Royal Derby Hospital Centre, Derby, United Kingdom; 5 Department of Surgery, Faculty of Medicine, Northern Border University, Arar, Saudi Arabia; National Institutes of Health, UNITED STATES

## Abstract

The heavy metals (HMs) lead and cadmium are persistent environmental pollutants capable of inducing ill-health in exposed individuals. One of the primary sites of accumulation and potential damage from HMs is bone, and we therefore examined the acute effects of lead and cadmium on human bone osteoblasts *in vitro* over a concentration range of 0.1 μM to 1mM, and for 3, 6, 12, 24, and 48 hour exposures. Incubation of osteoblasts with either lead or cadmium reduced cell viability in a concentrations and exposure durations dependent manner, as measured using MTT and LDH assays. Cytotoxicity was significant from 0.1 μM concentrations after 48 hour exposures. Both HMs damaged cellular bioenergetics with reductions of ATP production, mitochondrial complex activities, and aerobic respiration. There was a concomitant elevation of reactive oxygen species, with induction of redox stress measured as increased lipid peroxidation, and depleted cellular redox defense systems via reduced superoxide dismutase and catalase activity and cellular glutathione levels. Both HMs induced nuclear activation of Nrf2, presumably to increase transcription of antioxidant responsive genes to combat oxidative stress. Incubation of osteoblasts with HMs also compromised the secretion of procollagen type 1, osteocalcin, and alkaline phosphatase. Pre-incubation of osteoblasts with reduced glutathione prior to challenge with HMs lessened the cytotoxicity of the HMs, indicative that antioxidants may be a beneficial treatment adjunct in cases of acute lead or cadmium poisoning.

## Introduction

Heavy metals (HMs) such as lead, cadmium, mercury, and arsenic are worldwide environmental pollutants with multiple adverse health effects [1]. Humans may experience occupational or environmental exposure to HMs usually via inhalation, dermal, or ingestion routes, primarily via contaminated air, soil, water, or foodstuffs, respectively [2]. For some HMs, bone can be a primary target for HM accumulations and toxic damage. For example, bones may sequester and store lead, which can be subsequently remobilized during childhood or pregnancy, with neurotoxic or teratogenic properties, respectively [3–5]. Although higher bone mineral density (BMD) has been attributed to higher lead exposure in children [6], reduced BMD correlated with lead exposure in certain adult cohorts [7].

Kidney damage typifies environmental toxicity from cadmium exposures. However, cadmium also damages other tissues including lung, liver, and testis as well as exerting toxic effects upon bones. Bone softening (osteomalacia), reduced bone density (osteoporosis), increased bone fracture risk, and the development of Itai-Itai disease (cadmium-induced renal tubular osteomalacia) are clinical outcomes for humans that have experienced environmental exposure to cadmium [8–13].

The harmful effects of HMs may arise through organ toxicity such as cadmium-induced renal damage, or through secondary effects on metabolism, influencing vitamin D activation and calcium hemostasis [14–17]. Additionally, HMs may exert direct effects on bone cells, influencing bone formation and/or resorption.

Bone is essential for locomotion and skeletal function, with a principal function of provision of resistance to mechanical forces. The composition of bone is approximately 60% mineral crystals, 30% organic matrix, and 10% cells. There are four cell types: osteoblasts, osteoclasts, osteocytes, and bone lining cells [18]. Bone in not inert but a dynamic tissue that undergoes a continuous process of renewal through bone remodeling, via a balance between matrix synthesis by osteoblasts (of mesenchymal origin) and bone resorption by osteoclasts (of hematopoietic origin) [19,20]. An imbalance between matrix formation and resorption, results in net bone loss, as in cases of osteoporosis [21,22].

Osteoblasts are differentiated, cuboidal cells that line the bone surface and are responsible for bone matrix protein production; this includes the secretion of collagen (mainly type I) and non-collagenous proteins including osteocalcin, osteopontin, bone sialoprotein II, and alkaline phosphatase (ALP), crucial for bone mineralization [18].

To date, there is no level of exposure to lead that is without risk, indeed exposure to lead has been estimated to account for over half a million deaths in 2016 [23]. Lead accumulation in tissues promotes oxidative DNA damage, and causes anemia, in part through inhibition of the activity of δ-aminolevulinic acid dehydratase, an enzyme involved in the biosynthesis of heme [17, 24]. Cadmium exposure is also a major health concern, with cadmium classified as a human carcinogen [25,26]. In addition to their genotoxic effects, acute exposure to lead or cadmium triggers tissue damage and cell death via oxidative stress, depletion of protein thiols, mitochondrial dysfunction, and apoptosis [16,17,27–33]. Both heavy metals reduce activities of antioxidant defense enzymes and cellular glutathione levels, and promote lipid peroxidation in a number of target tissues [16,17,27–33].

Although lead and cadmium represent the two most abundant toxic metals and for which tissue toxicity has been extensively studied, their acute toxicity to bone cells has not been completely delineated. Furthermore, there is a need to establish the threshold lead or cadmium exposure that triggers osteoblast damage, and delineate the mechanisms of toxicity. To address this we have considered the cytotoxic effects of lead and cadmium after direct application to human osteoblasts *in vitro*. Additionally, we have considered the effects of both HMs upon cellular bioenergetics, the generation of redox stress, and whether HM administration influences osteoblast secretory functions.

## Material and methods

### 2.1 Chemicals and reagents

All chemicals, including the heavy metal solutions lead nitrate and cadmium dichloride, were purchased from Sigma (St. Louis, MO, USA), unless mentioned otherwise. For studies of oxygen consumption rates, Hank’s solution was prepared as described by Daunt *et al*. [34] and contained 5.6 mM KCl, 138 mM NaCl, 4.2 mM NaHCO_3_, 1.2 mM NaH_2_PO_4_, 2.6 mM CaCl_2_, 1.2 mM MgCl_2_, 10 mM HEPES (pH 7.4 with NaOH), and 0.1% (w/v) bovine serum albumin (BSA).

### 2.2 Cell culture

Osteoblasts were purchased from PromoCell (Heidelberg, Germany) and used at passages 4–5 for all studies. Cells were grown in Human Osteoblast Growth Medium and incubated at 37°C, 5% CO_2_ in a humidified incubator.

### 2.3 Cytotoxicity assessment using MTT and LDH assays

Osteoblasts were seeded at a density of 2 x 10^4^ cells/well, and grown to 90% confluence. Cells were then treated with Pb or Cd at concentrations of (0.1, 1, 10, 100, and 1000 μM) for 3, 6, 12, 24, or 48 hrs. The production of reduced MTT substrate as a measure of cell metabolic activity/viability was quantified spectrophotometrically at 590 nm according to the manufacturer’s protocol. All assay points were performed in triplicate. MTT absorbance values are expressed as percentages of control wells (defined as 100%).

For MTT assays performed in the presence of reduced glutathione, osteoblasts were prepared as before, but prior to treatment with HMs, cells were pre-incubated with 10 μM reduced GSH for 24 hours.

A commercially available kit was used to measure LDH levels, with methods followed according to the manufacturer’s protocol (Clontech, Mountain View, California, USA). Cells were seeded as for the MTT assay, and likewise incubated at the concentrations and for the durations detailed for the MTT assay. The release of LDH was measured spectrophotometrically at 490 nm using a plate reader ‘TopCount’ (Perkin Elmer, Ueberlingen, Germany). Positive control wells containing 2% Triton X-100 were used to provide a reference for total cell lysis. Cell cytotoxicity and the associated release of LDH was calculated as a percentage of the positive control.

### 2.4 Measurements of osteoblast proliferation

Osteoblast were seeded in 96 well plates at 2 x 10^4^ cells/well and then grown overnight. Cells were then treated with Pb (55 μM) or Cd (30 μM) for 12, 24, 48, 72, or 96 hrs. This concentration of HM reflects the estimated IC_50_ concentrations as determined using the MTT assay. 5-bromo-2’-deoxyuridine (BrdU) proliferation assays were performed according to the manufacturer’s guide (Millipore, Massachusetts, USA). Briefly, BrdU cell proliferation reagent was added at least 2 hr before the end of the incubation period with the HMs. After this HM incubation period, media was aspirated and 200 μl of fixing solution added per well, and samples incubated at room temperature for 30 minutes before aspiration. Well washing with a wash buffer was performed prior to the addition of 100 μL/well of diluted anti-BrdU monoclonal antibody, and incubation for 1 hour at room temperature. After well washing, goat anti-mouse IgG 100 μL/well was added, and the plate incubated for 30 minutes at room temperature. After further well washing, the plate was air dried. One hundred μL of peroxidase substrate was added to each well and the plate incubated in the dark for 30 minutes, and then the reaction stopped by pipetting 100 μL of acid stop solution to each well. The absorbance at 450 nm was measured using a microplate reader. All assay points were performed in triplicate, with control well values subtracted from test samples.

### 2.5 Measurements of intracellular ATP levels

Osteoblasts were seeded at 2 x 10^4^ cells/well and grown to 90% confluence, before treatment with Pb (55 μM) or Cd (30 μM) for 24 hrs. Levels of intracellular ATP were then measured according to a kit protocol (Abcam, Cambridge, UK). ATP levels, measured as luminescence, were detected by single photon counting with values quantified relative to control well readings.

### 2.6 Measurements of mitochondrial membrane potential

Osteoblasts were seeded in 24-well plates at 3 x 10^4^ cells/well. At confluence, cells were treated with Pb (55 μM) or Cd (30 μM). After 24 hrs, media were removed and cells incubated with a staining solution of mitotracker green (50 nM) at 37 °C. After 30 minutes, stained cells were retained in fresh phosphate buffered saline. Fluorescence was measured using a fluorescence microplate reader ‘TopCount’ (Perkin Elmer, Ueberlingen, Germany), using excitation and emission filters of 490 nm and 516 nm, respectively. Carbonyl cyanide-*4*-(trifluoromethoxy)phenylhydrazone (FCCP) was used as a positive control to trigger membrane uncoupling.

### 2.7 Measurements of mitochondrial complex activities

Osteoblasts were seeded at 4 x 10^4^ cells/well in six well plates, and incubated for 24 hrs with either Pb (55 μM) or Cd (30 μM) as determined using the MTT assay. A mitochondrial enriched fraction for complex I assay and cell lysate for complex III assay were prepared according to Spinazzi *et al*. [35]. Complex I activity was measured following the protocol of Janssen *et al* [36]. Complex III activity was assayed according to Spinazzi *et al*. [35]. Dichloroindophenol (DCIP) was used as the terminal electron acceptor.

### 2.8 Measurements of lactate production

Cells were seeded in 24-well plates at 5 x 10^4^ cells/well, and treated with Pb (55 μM) or Cd (30 μM) for 24 hrs. Cells were trypsinized, and then briefly spun at 1000 x g for 5 minutes. Pelleted cells were counted whereas the supernatant media was retained and assayed for lactate levels using a lactate assay kit (Biovision, Mountain View, California, USA) according to the manufacturer’s instructions. Lactate production was normalized to cell number and expressed as a percentage of control lactate production.

### 2.9 Measurements of oxygen consumption rate

The oxygen consumption rate (OCR) of osteoblast suspensions of known cell density was measured polarographically using Clark oxygen electrodes (Rank Brothers, Bottisham, UK), similar to a previous publication [37]. Cells were incubated with Pb (55 μM) or Cd (30 μM) for 24 hours. Cells were harvested by trypsinization, centrifuged and re-suspended in Hank’s solution and counted using a haemocytometer. The OCR was then assessed under basal conditions for 10 minutes before 2 μl of 6 mM azide was added to each chamber. The OCR was measured as the change in PO_2_ level over a 300-sec period with the azide slope measured 60 seconds after its addition. The majority of PO_2_ changes were linear under these conditions.

### 2.10 Measurements of reactive oxygen species

The relative levels of ROS generated in response to treatment with Pb or Cd were calculated based upon a 2,7-dichlorodihydrofluorescein diacetate (DCFDA) assay as described in a previous publication [38]. Osteoblast incubation with Antimycin A (10 mM for 30 min) was used as a positive control, with non-stained cells used for negative control values.

### 2.11 Measurements of catalase activity

Catalase (CAT) activity was assayed colorimetrically at 620 nm and expressed as μmoles H_2_O_2_ consumed/min/mg of protein using the method described by Singh *et al*. [39]. Assays were performed 24 hrs post-exposure to HMs. The reaction mixture (1.5 mL) contained 1.0 mL of 0.01M pH 7 phosphate buffer, 0.1 mL of tissue homogenate and 0.4 mL of 2M H_2_O_2_. The reaction was stopped by the addition of 2 mL of dichromate-acetic acid reagent.

### 2.12 Measurements of superoxide dismutase activity

Superoxide dismutase (SOD) activity measurements were based on SOD-mediated inhibition of the reduction of nitroblue tetrazolium to blue formazan by superoxide anions as described by Beauchamp and Fridovich [40]. Units of SOD activity were expressed in terms of mg of total protein.

### 2.13 Measurements of reduced glutathione

Reduced glutathione (GSH) was determined based upon the original method of Ellman as described by Ullah *et al*. [41]. Absorbance values were measured spectrophotometrically at 412 nm using a plate reader ‘TopCount’ (Perkin Elmer, Ueberlingen, Germany). The activity of GSH was expressed as nM reduced GSH/g tissue.

### 2.14 Measurements of lipid peroxidation

Thiobarbituric acid reactive substances (TBARS) were quantified as markers of lipid peroxidation [42]. Osteoblasts were treated with Pb (55 μM) or Cd (30 μM) (IC_50_ concentrations according to the MTT assay), and also at 10 μM for 24 hrs. Media was removed, cells were harvested, and then a TBARS assay performed according to Alam *et al*. [43].

### 2.15 Measurements of Nrf2 activation

Activation of Nrf2 was studied using a Nrf2 Transcription Factor Assay Kit (Abcam, Cambridge, UK). Osteoblasts were seeded in 75 cm^2^ flasks and grown to 90% confluence, before treatment with Pb (55 μM) or Cd (30 μM) for 24 hours. Nuclear extracts were prepared according to the assay kit protocol, protein concentrations determined using a Bradford assay, and then samples stored at -80 °C until required for assay. Assays for Nrf2 activation followed the manufacturer’s guidelines. Briefly, 40μl of binding buffer was added to each well of a 96 well assay plate, and then 15 μg of nuclear extract added in 10μl of lysis buffer. The plate was sealed and incubated at room temperature on a rocking platform for 1 hour, and then each well washed three times with wash buffer before addition of anti-Nrf2 primary antibody (100μl/well) and the plate then incubated for 60 minutes. Wells were washed before secondary antibody applied at room temperature for 1 hour. Finally, the developing solution was added (100μl/well) and plate incubated in the dark for 10 minutes before the addition of stopping solution. Well absorbance was read at OD 450nm (Perkin Elmer, Ueberlingen, Germany), with blank readings subtracted from final absorbance values. All assay points were performed in triplicate.

### 2.16 Effect of HMs on procollagen secretion

Osteoblasts were seeded at 4 x 10^3^ cells/well in 96-well plates and grown to 80–90% confluency. Cells were then treated with Pb (55 μM) or Cd (30 μM) for 24 hrs. Media was collected, sonicated, and procollagen type I C-Peptide (PIP) quantified using an ELISA kit (Takara Shuzou, Japan), with PIP concentrations interpolated from a standard curve. Cells were homogenized in phosphate buffered saline containing 0.5% Triton X-100, 1 mM ethylenediaminetetraacetic acid, and 1 mM phenylmethylsulfonyl fluoride, with procollagen content determined as for media and normalized to cell number.

### 2.17 Effect of HMs on osteocalcin secretion

Osteoblasts were seeded at 4 x 10^3^ cells/well in 96-well plates and grown to 80–90% confluency. Cells were then treated with Pb (55 μM) or Cd (30 μM) for 3, 6, 12, 24 or 48 hrs. Media were collected and the levels of osteocalcin determined using a sandwich enzyme-linked immunosorbent assay (ELISA) kit (Takara Shuzou, Japan), with osteocalcin concentrations interpolated from a standard curve.

### 2.18. Effect of HMs on alkaline phosphatase secretion

Osteoblasts were seeded at 4 x 10^6^ cells/well in 24-well plates and grown to 80–90% confluency. Cells were treated with Pb (55 μM) or Cd (30 μM) for 3, 6, 12, 24 and 48 hrs. Media was collected, and alkaline phosphatase (ALP) activity evaluated using a commercially available kit following the manufacturer’s protocol (Abcam, Cambridge, UK). A *p*-nitrophenyl phosphatase assay was used to measure ALP activity within media, and quantified spectrophotometry at 405 nm using a TopCount plate reader (Perkin Elmer, Ueberlingen, Germany).

### 2.19 Statistical analysis

All statistical analyses were performed using PRISM 5 (GraphPad Software Inc., San Diego, California, USA). One way analysis of variance (ANOVA) test with Dunn’s multiple comparisons post-test or unpaired student t-test were used for comparisons of the different groups data. Statistical significance was defined as *p <* 0.05.

## Results

### 3.1 Pb and Cd are cytotoxic to human osteoblasts

**Fig 1:**
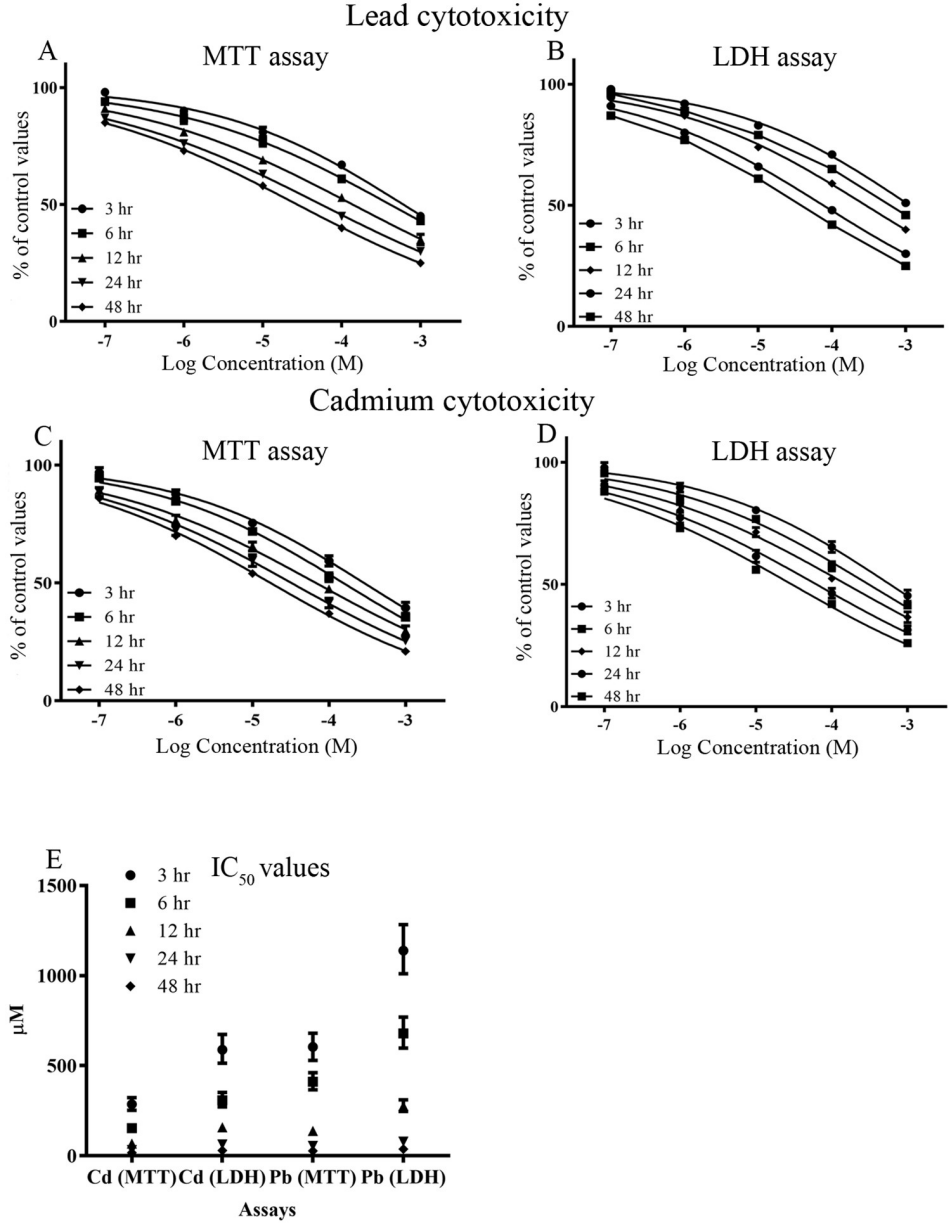
Lead and cadmium cytotoxicity to human osteoblasts *in vitro*. Human osteoblasts were treated with lead or cadmium over a concentration range of 0.1–1000 μM and for 3–48 hours and the level of cell metabolic activity and viability determined by MTT and LDH assays **(A-D)**. The concentrations of lead or cadmium that produced 50% inhibition of metabolic activity or cell viability were determined **(E)**. Data points are representative of 9 independent experiments.

Human osteoblasts were incubated with the HMs, lead (Pb) or cadmium (Cd) over a broad concentration range of 0.1 μM to 1 mM, and the level of cytotoxicity quantified after 3, 6, 12, 24, and 48 hours using MTT and LDH assays. Both Pb and Cd were cytotoxic to osteoblasts in a concentration and duration dependent manner (Fig 1A–1D, Table 1, and S1 and S2 Tables). Even at 0.1 μM, Pb and Cd were significantly cytotoxic to osteoblasts 48 hours post-treatment (*p* = 0.035 and 0.023, respectively) (Fig 1A–1D and S1 and S2 Tables). Cd was more cytotoxic than Pb with lower IC_50_s at all tested time points (Fig 1E, Table 1, and S1 and S2 Tables). That these HMs induced a cytotoxic response was further confirmed via a BrdU cell proliferation assay that demonstrated that neither agent stimulated osteoblast proliferation (results not included).

**Table 1:** IC_50_ values (μM) for lead and cadmium cytotoxicity to human osteoblasts *in vitro*.

Assay	Duration of exposure (hr)				
	3	6	12	24	48
Pb (MTT)	604.2 (680.6–530.3)	411.2 (461.9–366.1)	137.8 (150.8–125.9)	54.9 (58.9–51.1)	27.8 (37.7–20.5)
Pb (LDH)	1139.2 (1283.4–1011.1)	678.6 (769.8–598.2)	276.5 (310.9–245.9)	78.8 (84.0–73.8)	37.4 (39.3–35.7)
Cd (MTT)	286.1 (323.5–253.3)	153.2 (167.8–139.9)	67.5 (75.4–60.3)	32.3 (36.7–28.5)	17.3 (18.5–16.1)
Cd (LDH)	588.7 (673.9–514.2)	309.1 (352.6–271.4)	157.7 (178.5–139.2)	63.0 (72.3–55.0)	29.9 (33.0–27.0)

### 3.2 Pb and Cd damage osteoblast bioenergetics

**Fig 2:**
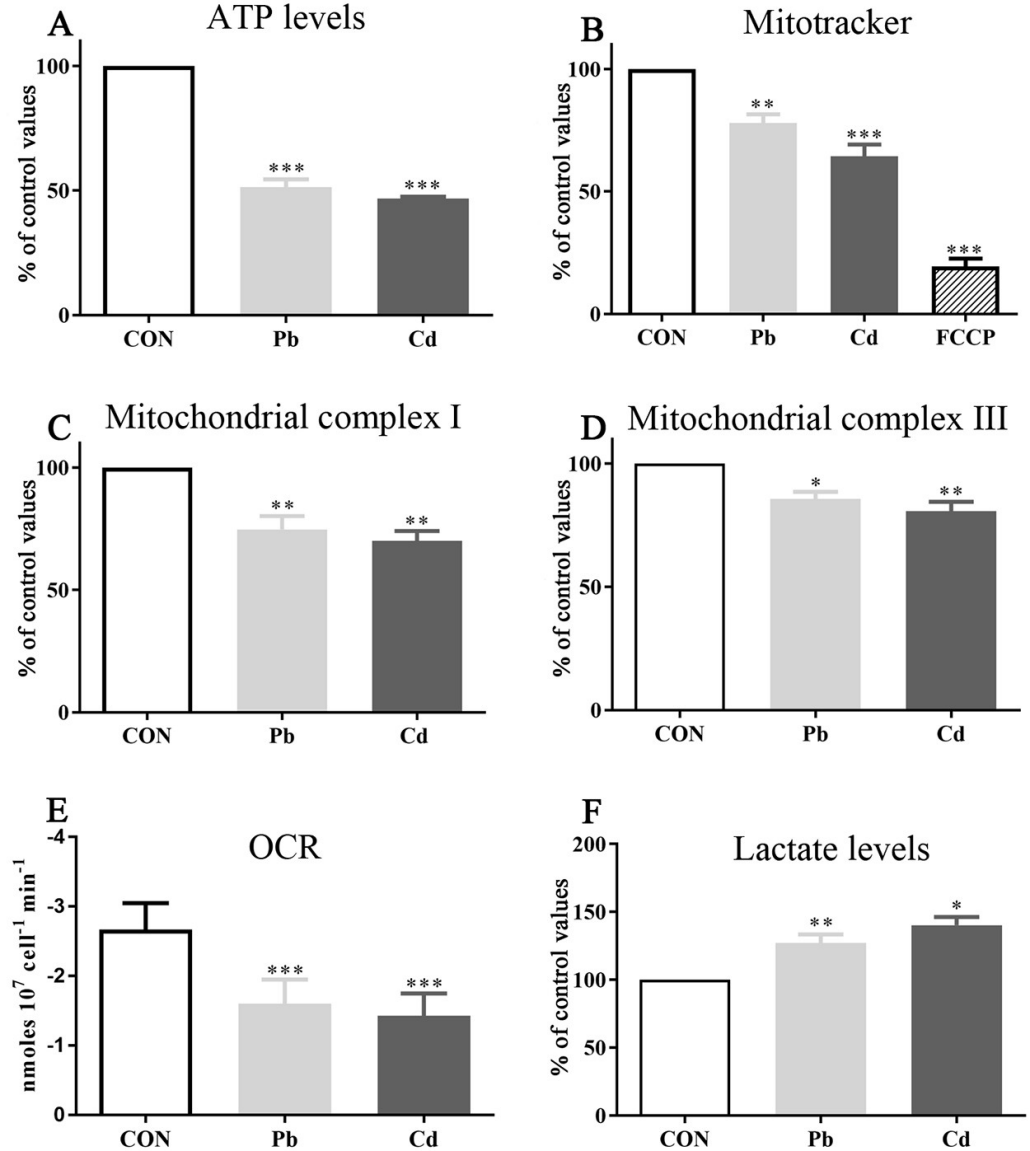
The effect of lead or cadmium on human osteoblast bioenergetics. Human osteoblasts were treated with lead (Pb) (55 μM) or cadmium (Cd) (30 μM), and the production of cellular ATP **(A)**, mitochondrial membrane potential **(B)**, mitochondrial complex I activity **(C)**, mitochondrial complex III activity **(D)**, oxygen consumption rate (OCR) **(E)**, and lactate levels **(F)** quantified. Significant differences from controls are marked with asterisks. Data are representative of 6–9 independent experiments. For * = *p*-value < 0.05, ** = *p*-value < 0.01, *** = *p*-value < 0.0001.

The investigation of cellular bioenergetics provided an additional independent evaluation of osteoblast viability, and an insight to the molecular mechanism of cytotoxicity. Treatment of osteoblasts with Pb or Cd for 24 hours at concentrations of 55 μM and 30 μM, respectively (their IC_50_ values as determined using the MTT assay), significantly reduced intracellular ATP levels to 58 and 54% of controls, respectively (*p* = <0.0001) (Fig 2A). Since the production of ATP is governed by oxidative phosphorylation linked to the mitochondrial electron transport system, a determination of the mitochondrial membrane potential, and activity assays for the mitochondrial enzymes, complex I and complex III, were undertaken. A 24 hr exposure to Pb or Cd significantly reduced mitochondrial membrane potential (*p* < 0.0001) (Fig 2B), mitochondrial complex I activity (*p* = 0.0035) (Fig 2C), and mitochondrial complex III activity (*p* = 0.0063) (Fig 2D). Cellular bioenergetics were also assessed using a polarographic assay of oxygen consumption rates (OCRs). Pb or Cd significantly lowered osteoblast OCRs (*p* < 0.0001) (Fig 2E), and, consistent with an inhibition of aerobic metabolism, both HMs triggered a significant increase in the production of lactate (*p* = 0.0037) (Fig 2F).

### 3.3 Pb and Cd damage osteoblasts via generation of redox stress

**Fig 3:**
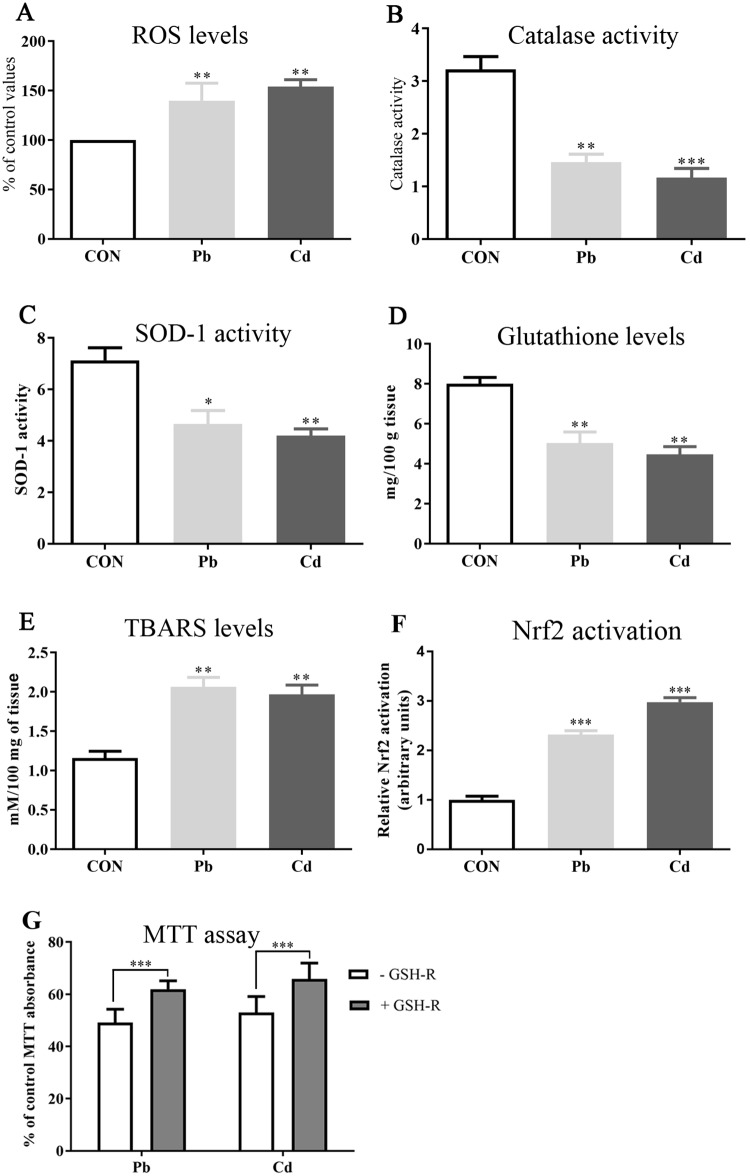
The effect of lead or cadmium on redox status of human osteoblasts. Human osteoblasts were treated with lead (Pb) (55 μM) or cadmium (Cd) (30 μM) for 24 hours, and the production of ROS **(A)**, catalase activity **(B)**, superoxide dismutase-1 (SOD-1) activity **(C)**, intracellular reduced glutathione levels **(D)**, production of thiobarbituric acid reactive substances (TBARS) **(E)**, and activation of Nrf2 **(F)** quantified. Human osteoblasts were treated with reduced glutathione prior to treatment with lead or cadmium, and the level of cytotoxicity determined using an MTT assay **(G)**. Significant differences from controls are marked with asterisks. Data are representative of 5–9 independent experiments. For * = *p*-value < 0.05, ** = *p*-value < 0.01, *** = *p*-value < 0.0001.

To assess redox stress, osteoblasts were incubated with Pb (55 μM) or Cd (30 μM) at their MTT IC_50_ concentrations. Measurements of reactive oxygen species (ROS) were conducted *in situ* using a dichlorofluorescin diacetate (DCFDA) assay. Pb or Cd significantly increased cellular ROS production (*p* = 0.0023) by 39 and 54%, respectively (Fig 3A).

We then investigated the activity of the cellular redox regulating enzymes, catalase and superoxide dismutase (SOD), and the cellular levels of reduced glutathione. Pb or Cd significantly decreased catalase activity to 45% and 36% of controls, respectively (*p* = 0.0006); SOD activities to 65% and 59% of controls, respectively (*p* = 0.0066), and glutathione levels to 63% and 56% of controls, respectively, (*p* = 0.0024) (Fig 3B–3D).

The redox stress triggered by Pb or Cd induced an increase of the levels of lipid peroxidation products as thiobarbituric acid reactive substances (TBARS) by 78% and 69% of control levels, respectively, (*p* = 0.002) (Fig 3E).

Induction of cellular redox stress by Pb or Cd was consistent with significant activation of the nuclear (redox regulatory) transcription factor, Nrf2, by 2.32- and 2.98-fold, respectively (*p* < 0.0001) (Fig 3F). To demonstrate that Pb or Cd induced redox stress contributed to reduced cell viability, osteoblasts were pre-incubated with reduced glutathione (GSH) prior to challenge with Pb or Cd. Pre-incubation with GSH significantly lowered the cytotoxic effects of Pb (*p* = 0.0004) or Cd (*p* < 0.0001) (Fig 3G).

### 3.4 Pb and Cd toxicity affects human osteoblast secretory functions

**Fig 4:**
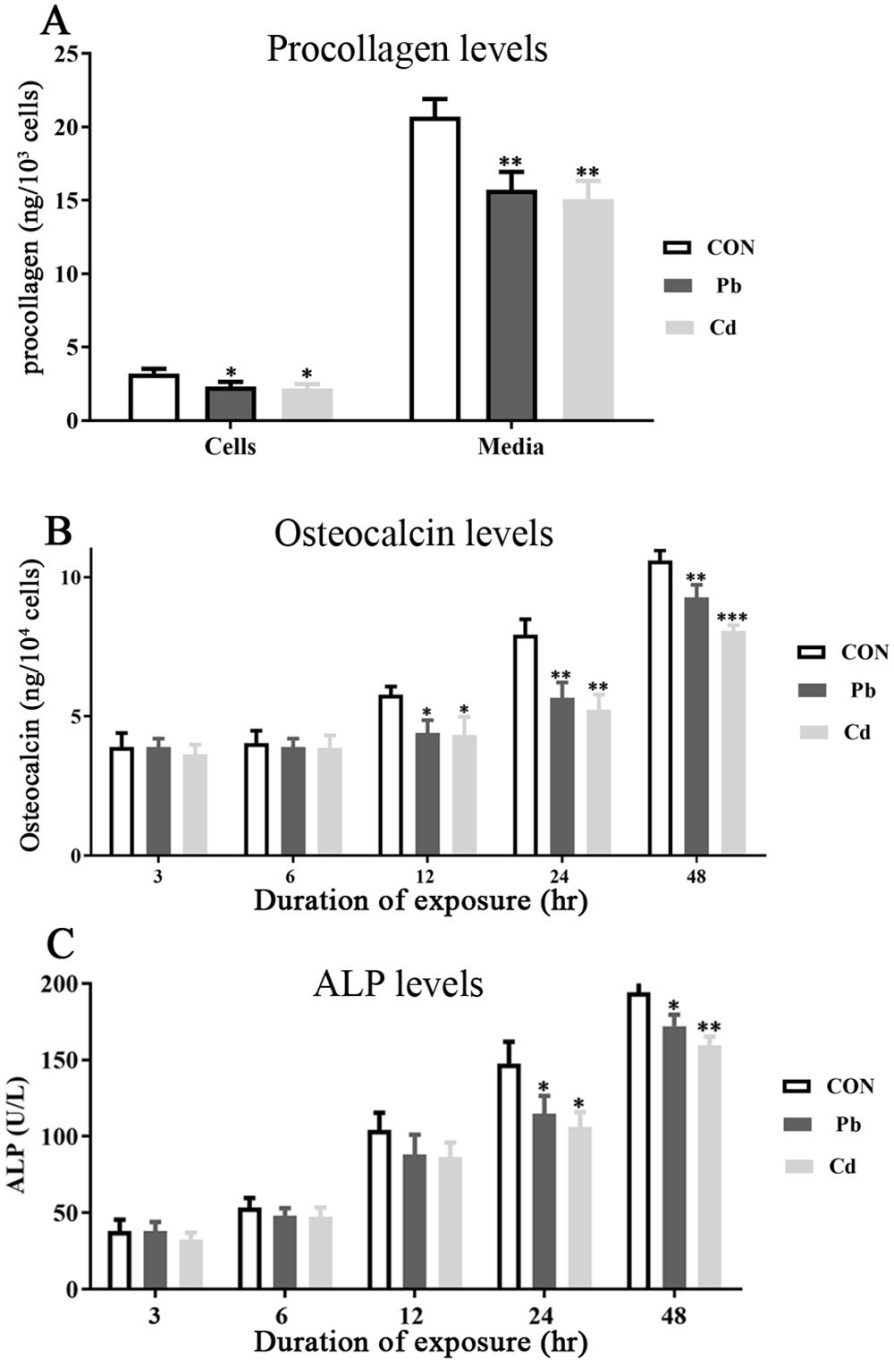
The effect of lead or cadmium (Cd) on human osteoblast secretions. Human osteoblasts were treated with lead (Pb) (55 μM) or cadmium (Cd) (30 μM), and the level of procollagen type I peptide within the cells and released into the culture media quantified **(A)**. At 3, 6, 12, 24, and 48 hours post-treatment with lead or cadmium, the levels of osteocalcin **(B)** and ALP **(C)** secreted into the culture media were quantified. Significant differences from controls are marked with asterisks. Data are representative of 5 independent experiments. For * = *p*-value < 0.05, ** = *p*-value < 0.01, *** = *p*-value < 0.0001.

The effect of HMs on the osteoblast production of procollagen type I peptide and osteocalcin were quantified. The secretion of procollagen was significantly reduced in cells and also that secreted into the media after a 24 hour incubation with Pb (55 μM) (*p* = 0.0126) or Cd (30μM) (*p* = 0.0025) (Fig 4A).

The secretion of osteocalcin was quantified at 3, 6, 12, 24, and 48 hour time points after treatment of osteoblasts with Pb (55 μM) or Cd (30μM). After 12, 24, or 48 hours the production of osteocalcin was significantly reduced by Pb or Cd by 18 and 20% (*p* = 0.019), 26 and 30% (*p* = 0.002), and 13 and 24%, (*p* = 0.0004), respectively (Fig 4B).

The secretion and activity of ALP was quantified at 3, 6, 12, 24, and 48 hour time points after treatment of osteoblasts with Pb (55 μM) or Cd (30μM). After 24 and 48 hours ALP was significantly reduced by Pb or Cd by 26 and 30% (*p* = 0.026), and 18 and 20% (*p* = 0.0062), respectively (Fig 4C).

## Discussion

### 4.1 Lead and cadmium are cytotoxic to human osteoblasts

Our study examined the cytotoxic effects of two widely spread and encountered HMs, lead and cadmium, and considered their direct effects on human osteoblasts *in vitro*. The four cell types resident within bone: osteoblasts, osteoclasts, osteocytes, and bone lining cells exist in a dynamic interaction and collectively form a basic multicellular unit (BMU) [18,20]. However, for ease of study, and in order to delineate cell specific effects, analysis of a single homogeneous population of cells is required. Hence, our approach was to utilize a human osteoblast cell line rather than extraction of primary cells. This ensured the use of phenotypically similar cells, avoided any species-specific responses, and has also been shown to provide results consistent with primary cells [44].

Lead is toxic to a number of bodily systems, including bone. There are no safe levels of lead exposure [23], with initial clinical symptomology that includes kidney dysfunction at a blood lead level (BLL) of 5–10 μg/dL (0.24–0.48 μM). A spectrum of health impact is observed above 11 μg/dL, with cognitive impairment at 40–79 μg/dL, neuropathy at >80 μg/dL (≈4 μM), and encephalopathy at 100–120 μg/dL (≈6 μM) [45]. Since levels of human intoxication from lead exposure are correlated with BLL, we examined a broad concentration range that included symptomatic BLL concentrations (0.1–1 μM), as well as gross intoxication (>10 μM). Noteworthy is the recent demonstration that bone lead levels significantly correlate with past maximum and current BLLs [46].

The accumulation of cadmium is reflected by its 24 hour urinary excretion, and typically reported as either nmol of cadmium excretion/day or as creatine-adjusted urinary cadmium levels. For smelter workers occupationally exposed to lead and cadmium, blood cadmium levels up to 0.145 μmol/L, and BLLs of 1–3.7 μmol/L have been reported [15]; comparable to our 0.1–1 μM exposure measurements. Furthermore, by using the same exposure concentration range for cadmium as that employed for lead, we were able to make direct toxicity comparisons between the two HMs, and establish if similar mechanisms of toxicity existed at a given exposure concentration.

Although BLL concentrations of >10 μM would be considered overdose levels, exposure of cells *in vitro* at relatively high concentrations is used to model more chronic and cumulative exposure within a limited period of study. Furthermore, HMs including lead and cadmium are non-degradable, and have a slow rate of elimination (blood lead has a half-life of about 40 days in humans, cadmium an elimination half-life of 20–30 years); they therefore can progressively accumulate in tissues such as bone that sequesters HMs.

Both HMs were cytotoxic to osteoblasts in a concentrations and exposure durations dependent manner, as assessed using a MTT assay (Fig 1, Table 1, S1 and S2 Tables). The tetrazolium (MTT) assay is based upon the ability of NAD(P)H-dependent cellular oxidoreductase enzymes to reduce MTT to formazan, but may not discriminate between cytostatic and cytotoxic agents. Hence, to confirm cytotoxicity of the HMs, production of extracellular LDH was also quantified. The liberation of LDH relates to a loss of the cell membrane integrity experienced at cell death. The LDH concentration curves for cell cytotoxicity mirrored those generated from MTT assays (refer to Fig 1), confirming that lead or cadmium induced cell death with increasing concentration and duration of exposure. The threshold concentration for cell death was 0.1 μM for both lead and cadmium, after a 48 hour and 24 hour exposure, respectively. At 1 μM, cytotoxic effects were evident after 6 hours for lead and 3 hours for cadmium (S1 and S2 Tables); concentrations comparable with BLLs, or cadmium blood levels experienced by occupationally or environmentally exposed individuals [15].

Collectively, cadmium was more cytotoxic than lead, with lower IC_50_s at all tested time points (refer to Table 1, S1 and S2 Tables). The cytotoxicity of lead or cadmium to either osteoblast-like cell lines, or primary osteoblasts is via induction of apoptosis and necrosis [47–49]. Even lower IC_50_s (higher sensitivity) have been reported using rat primary osteoblasts (IC_50_ of 2 μM for cadmium) [49], which may reflect species-specific effects as well as improved responsiveness of primary cells.

### 4.2 Lead and cadmium exposure impairs human osteoblast cellular bioenergetics and generates redox stress

Our study demonstrated that lead or cadmium when applied to cells at 55 μM and 30 μM, respectively, damaged osteoblast bioenergetics. A reduction of mitochondrial membrane potential was evident, with disruption and inhibition of mitochondrial complex protein activities; components of the electron transport chain (ETC) crucial for cellular (aerobic) respiration (Fig 2). A loss of electron transport and coupling to oxidative phosphorylation resulted in a shift to anaerobic metabolism, with reduced oxygen consumption, increased lactate production, and reduced ATP production (Fig 2).

The ability of toxic agents to induce damage to mitochondria and nullify ATP production is a common mechanism of cellular damage, and contributes to cellular redox stress and induction of apoptosis [50–56]. There is normally leakage of protons from the ETC into the mitochondrial matrix. These protons can combine with oxygen molecules to form reactive oxygen species (ROS); a process increased under pathological conditions [53,57]. ROS are able to damage DNA, proteins, and lipids; the latter consistent with an increase in lipid peroxidation (Fig 3E). The generation of ROS may be further self-propagating since ROS also damage mitochondria and thereby promote membrane permeation, and potentially more ROS production and accumulation of HMs [58].

ROS levels within osteoblasts increased in response to a 24 hour exposure to lead (55 μM) or cadmium (30 μM), with cellular redox stress exacerbated by a reduction in the activities of superoxide dismutase (SOD) and catalase, as well as diminished cellular glutathione levels (Fig 3). SOD catalyzes the dismutation of the superoxide radical to form either molecular oxygen or hydrogen peroxide. Hydrogen peroxide can be decomposed to water and molecular oxygen via the action of catalase. Glutathione is the major cellular thiol able to buffer and resist redox stress. Hence, lead or cadmium induced redox stress will be compounded once SOD and catalase activity are compromised and glutathione depleted; as collectively they comprise major elements of cellular redox defense. Consistent with our *in vitro* data, biomarkers of redox stress have also been detected in blood after occupational exposure to lead or cadmium [59,60]. Cells are able to resist the detrimental effects of xenobiotic and redox stress promoters via activation of the transcription factor, nuclear factor E2-related factor 2 (Nrf2) [61,62]. We detected significantly increased nuclear Nrf2 as a response to toxic levels of lead or cadmium (Fig 3F). Upon appropriate cellular stimulus (including redox stress), Nrf2 translocates from the cytoplasm to the nucleus to promote transcription of a vast array of cytoprotective genes, including those for antioxidant and detoxificating enzymes [61,62]. At present we can only speculate that the increased Nrf2 activity we have observed is to mitigate redox stress [63,64], and a comprehensive determination of the transcriptional and translational changes induced after Nrf2 activation are still required.

### 4.3 HMs decrease the secretory output from osteoblasts

The organic matrix of bone is primarily (≈90%) comprised of collagenous proteins (predominantly type I collagen), as well as non-collagenous proteins such as osteocalcin and ALP secreted by osteoblasts. This prompted us to consider the ability of osteoblasts to secrete type I procollagen, osteocalcin, and ALP.

Treatment of osteoblasts with either lead or cadmium impaired the secretion of these proteins (Fig 4). Collagen fibers, formed from secreted procollagen, contribute to the structural strength of bone [21]. A toxic exposure to lead or cadmium that evokes a reduction of collagen production may reduce bone strength, a process consistent with the increased fracture risk associated with HM exposures [12,13].

Osteocalcin promotes bone mineralization and density, but also has an emerging role as a bone-derived hormone that influences energy metabolism, and brain development and cognition [65]. Notably, even at relatively low BLL, lead exposure in children results in cognitive impairment and IQ deficits [4]. Given the growing appreciation of the importance of osteocalcin in neuronal development, reduced osteocalcin production as a consequence of elevated lead could impact on bone strength as well as contribute to the effects of lead on neurodevelopment.

ALP functions to degrade phosphate-containing compounds, releasing phosphate ions within the bone matrix vesicles to promote matrix crystallization. Reduced ALP levels and associated activity would similarly be expected to limit bone strength and integrity.

### 4.4 Management of lead or cadmium exposure through treatment of redox stress

The current treatment strategy for lead poisoning is via administration of chelating agents [66,67]. This may be of acute benefit, with treatment of lead-exposed children with the chelating agent, succimer, able to reduce BLL after 1 week, but was without effect on BLL 1 year after treatment, indicative of remobilization of lead from stores such as bone [68]. Thus, additional acute, subacute, and chronic treatment options are needed to support current therapies. Since the cytotoxicity to osteoblasts from lead or cadmium exposure was mediated in part by damage to cellular bioenergetics and induction of redox stress, agents able to mitigate redox stress may be useful for cellular preservation. This was tested by pre-incubation of osteoblasts with reduced glutathione prior to exposure to HMs, and this improved cell viability (Fig 3F). Other studies have also proposed the benefits of dietary supplementation as a means to resist acute or subacute lead or cadmium induced redox stress *in vivo* [67,69], although whether this represents a useful chronic treatment has not yet been validated. Additionally, given the impact of these HMs on cellular bioenergetics, interventions that provide alternative respiratory substrates, such as β-hydroxybutyrate could prove useful therapeutic interventions if able to bypass or ameliorate HM-induced mitochondrial protein inhibition.

## Conclusion

**Fig 5:**
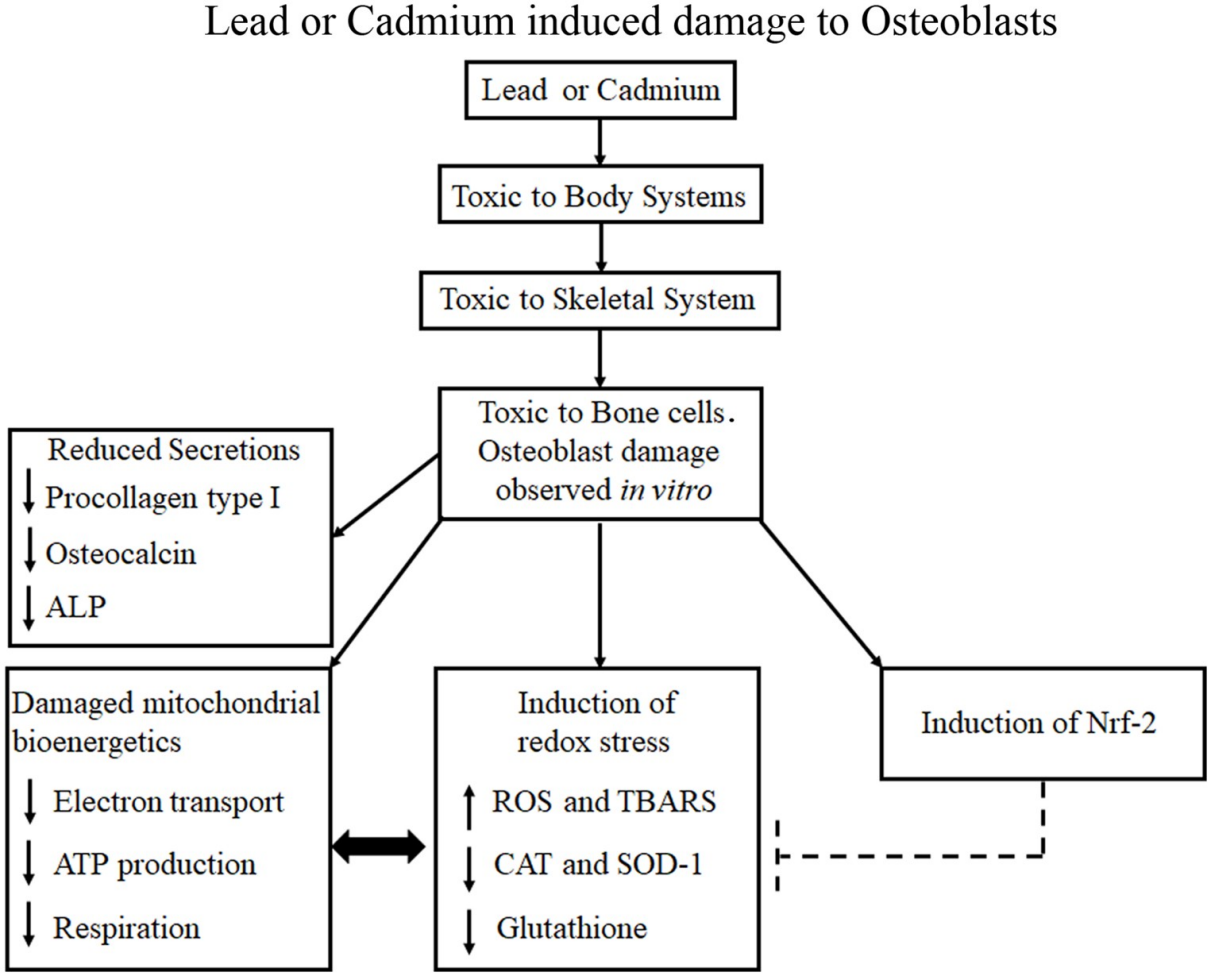
Toxic effects of lead or cadmium to human osteoblasts. The heavy metals (HMs) lead and cadmium were toxic to human osteoblasts *in vitro*. Both HMs damaged cellular bioenergetics; with reduced mitochondrial complex activities, ATP production, and aerobic respiration. Both HMs induced cellular redox stress via increased production of reactive oxygen species (ROS) with associated elevation of lipid peroxidation as thiobarbituric acid reactive substances (TBARS). Both HMs limited cellular redox defence via a lowering of catalase (CAT) and superoxidase dismutase-1 (SOD-1) activities, and reduced glutathione levels. Damaged mitochondria can liberate more ROS that in turn will further damage mitochondria (↔). The transcription factor Nrf2 was activated in response to HMs, and presumably acts to mitigate some of the mitochondrial and redox damage via upregulation of antioxidant genes. HM damaged osteoblasts displayed functional deficits with reduced secretion of procollagen type I, osteocalcin, and alkaline phosphatase (ALP).

To conclude, the environmental and occupational pollutants lead and cadmium, damage cellular bioenergetics of human osteoblasts, and induce redox stress via ROS generation and limiting the effectiveness of the cellular antioxidant defense systems. Osteoblast secretory functions were significantly reduced in parallel with the HM-induced bioenergetics disruption and oxidative damage (Fig 5). Since the induction of redox stress is one of the common mechanisms by which these agents induce cellular damage and death, administering antioxidants may provide a useful adjunct to support the currently employed chelation therapy.

## Supporting information

S1 Table Cytotoxic effect of lead to human osteoblasts *in vitro*. (DOCX)

S2 Table Cytotoxic effect of cadmium to human osteoblasts *in vitro*. (DOCX)
