# Supplementary material for: The heavy metals lead and cadmium are cytotoxic to human bone osteoblasts via induction of redox stress
Source: PLoS One. 2019 Nov 22;14(11):e0225341. doi: 10.1371/journal.pone.0225341 (PMC6874340; doi:10.1371/journal.pone.0225341)
Supplement: S1 Table — (DOCX) [file pone.0225341.s001.docx]

|  | Duration of exposure | | | | | | | | | |
| --- | --- | --- | --- | --- | --- | --- | --- | --- | --- | --- |
| Conc  (µM) | 3 hr | | 6 hr | | 12 hr | | 24 hr | | 48 hr | |
|  | MTT | LDH | MTT | LDH | MTT | LDH | MTT | LDH | MTT | LDH |
| 0.1 | 98.3 ^n^  ±0.98 | 98.2 ^n^  ±0.98 | 94.5 ^n^  ±1.20 | 96.5 ^n^  ±1.20 | 91.4 ^n^  ±1.40 | 94.5 ^n^  ±1.40 | 87 ^n^  ±0.97 | 91.9 ^n^  ±0.97 | 85 ^a^  ±1.20 | 87.2 ^a^  ±0.34 |
| 1 | 90.5 ^n^  ±1.20 | 92.3 ^n^  ±1.20 | 87.4 ^a^  ±2.40 | 89.3 ^a^  ±2.40 | 81.3 ^a^  ±1.20 | 85.4 ^a^  ±1.20 | 76 ^a^  ±1.30 | 82.7 ^a^  ±1.30 | 73 ^b^  ±1.40 | 77.4  ±0.97 |
| 10 | 81.4  ±2.10 | 83.1 ^a^  ±2.10 | 77.6 ^a^  ±2.10 | 82.3  ±2.10 | 69.5 ^b^  ±1.70 | 72.7 ^b^  ±1.70 | 63 ^b^  ±1.70 | 66.6 ^b^  ±1.70 | 58 ^b^  ±0.98 | 61.4 ^c^  ±1.20 |
| 100 | 67.3 ^b^  ±1.20 | 71.5 ^b^  ±1.20 | 61.3 ^c^  ±1.80 | 67.4 ^b^  ±1.80 | 53.2 ^c^  ±1.20 | 59.5 ^c^  ±1.20 | 45 ^c^  ±1.30 | 48.4 ^c^  ±1.30 | 40 ^c^  ±0.97 | 42.7 ^c^  ±0.98 |
| 1000 | 45.2 ^c^  ±1.20 | 51.4 ^c^  ±1.20 | 43.4 ^c^  ±1.20 | 46.6 ^c^  ±1.20 | 35.8 ^c^  ±2.20 | 40.2 ^c^  ±2.20 | 30 ^c^  ±1.20 | 30.3 ^c^  ±1.20 | 25 ^c^  ±1.50 | 25.7 ^c^  ±1.30 |

**S1 Table. Cytotoxic effect of lead to human osteoblasts *in vitro*.**

Values are relative to control levels (100%) for MTT and LDH assays.

n, denotes a *p*-value > 0.05; a, denotes a *p*-value < 0.05; b, denotes a *p*-value < 0.01, c, denotes a *p*-value < 0.001.
