# Supplementary material for: The heavy metals lead and cadmium are cytotoxic to human bone osteoblasts via induction of redox stress
Source: PLoS One. 2019 Nov 22;14(11):e0225341. doi: 10.1371/journal.pone.0225341 (PMC6874340; doi:10.1371/journal.pone.0225341)
Supplement: S2 Table — (DOCX) [file pone.0225341.s002.docx]

**S2 Table. Cytotoxic effect of cadmium to human osteoblasts *in vitro*.**

|  | Duration of exposure | | | | | | | | | |
| --- | --- | --- | --- | --- | --- | --- | --- | --- | --- | --- |
| Conc  (µM) | 3 hr | | 6 hr | | 12 hr | | 24 hr | | 48 hr | |
|  | MTT | LDH | MTT | LDH | MTT | LDH | MTT | LDH | MTT | LDH |
| 0.1 | 96.8^n^  ±2.10 | 97.8 ^n^  ±2.10 | 94.5 ^n^  ±0.98 | 95.4 ^n^  ±0.98 | 89.4 ^n^  ±1.20 | 91.3 ^n^  ±1.20 | 88.6^a^  ±1.9 | 90.7 ^n^  ±1.9 | 86.6 ^a^  ±0.96 | 88.3 ^a^  ±0.97 |
| 1 | 87.6 ^a^  ±1.80 | 89.6 ^n^  ±1.80 | 84.7 ^a^  ±1.70 | 84.6 ^a^  ±1.70 | 76.5b  ±2.30 | 80.4 ^a^  ±2.30 | 72.5^b^  ±2.2 | 77.3 ^b^  ±2.2 | 70.3 ^b^  ±0.68 | 73.3 ^b^  ±0.98 |
| 10 | 75.5 ^b^  ±1.50 | 80.4 ^a^  ±1.50 | 71.9 ^b^  ±1.40 | 76.8 ^b^  ±1.40 | 65.5 ^b^  ±1.90 | 71.5 ^b^  ±1.90 | 59.5^c^  ±2.5 | 61.5 ^c^  ±2.5 | 54.2 ^c^  ±1.10 | 56.9 ^c^  ±1.10 |
| 100 | 59.4 ^c^  ±2.20 | 65.3 ^c^  ±2.20 | 52.5 ^c^  ±1.90 | 57.4 ^c^  ±1.90 | 47.5 ^c^  ±1.50 | 52.6 ^c^  ±1.50 | 41.5 ^c^  ±2.0 | 46.4 ^c^  ±2.0 | 37.7 ^c^  ±0.87 | 42.6 ^c^  ±0.98 |
| 1000 | 39.5 ^c^  ±2.30 | 45.5 ^c^  ±2.30 | 35.5 ^c^  ±1.20 | 41.5 ^c^  ±1.20 | 29.7 ^c^  ±2.20 | 36.5 ^c^  ±2.20 | 25.45 ^c^  ±1.7 | 31.4 ^c^  ±1.7 | 21.8 ^c^  ±0.57 | 26.4 ^c^  ±0.68 |

Values are relative to control levels (100%) for MTT and LDH assays.

n, denotes a *p*-value > 0.05; a, denotes a *p*-value < 0.05; b, denotes a *p*-value < 0.01, c, denotes a *p*-value < 0.001.
